# Characterization of adult hippocampal neurogenesis in adult and aged genetically diverse mice

**DOI:** 10.1007/s11357-025-01749-9

**Published:** 2025-06-17

**Authors:** Adele E. Finch, Avery D. McNamara, Kristen D. Onos, Kelly J. Keezer, Gareth R. Howell, Ashley E. Webb

**Affiliations:** 1 Department of Neuroscience, Brown University, Providence, RI USA; 2 Department of Molecular Biology, Cell Biology, and Biochemistry, Brown University, Providence, RI USA; 3 The Jackson Laboratory, Bar Harbor, ME USA; 4 The Buck Institute for Research On Aging, Novato, CA USA; 5 Graduate School of Biomedical Sciences, Tufts University, Boston, MA USA; 6 Graduate School of Biomedical Sciences and Engineering, University of Maine, Orono, USA

**Keywords:** Adult hippocampal neurogenesis, Genetic diversity, Mouse models, Brain aging

## Abstract

Adult hippocampal neurogenesis—the generation of new neurons in the adult brain—declines with age, contributing to cognitive deficits in aging. While the majority of mammalian studies on neurogenesis have utilized inbred mouse strains, these models do not fully capture the genetic diversity of humans, limiting the translational relevance of their findings. The Diversity Outbred (DO) mouse model, a genetically heterogeneous population, provides a promising alternative to traditional inbred strains. In this study, we investigated how genetic diversity influences hippocampal neurogenesis by comparing neurogenesis in adult and aged Diversity Outbred (DO) mice with the commonly used C57BL/6J inbred strain. While both strains exhibited a decline in neurogenesis with age, DO mice showed significantly lower levels of neurogenesis compared to C57BL/6J mice, even in young adults. Additionally, we observed that the wild-derived CAST/EiJ strain, one of the eight founder strains in the DO model, contributed to this reduction in neurogenesis. Our findings highlight the importance of genetic diversity in neurogenesis research and suggest that the DO model may better represent human genetic diversity associated with age-related decline in neurogenesis.

## Introduction

Adult hippocampal neurogenesis, the process by which new neurons are generated in the adult brain, persists throughout life in mice and rats [1–3]. However, neurogenesis starkly declines with age, contributing to cognitive defects that occur both during healthy aging in rodents and in mouse models of neurodegenerative disease [3, 4]. Hippocampal neurogenesis has also been observed to decrease with age in humans and is further exacerbated by neurodegenerative disease [4–7]. Thus, targeting neurogenesis has arisen as an enticing therapeutic strategy for mitigating age-associated cognitive decline.

Most mammalian studies on adult neurogenesis utilize inbred mouse strains which offer several advantages, including minimization of genetic variability within cohorts and improved experimental reproducibility. However, the genetic homogeneity of these mice fails to capture the genetic diversity of humans, severely limiting the generalizability of translating experimental findings in these mice to the clinical setting. This is particularly apparent in the context of complex neurodegenerative diseases, which further emphasizes the value in prioritizing genetic complexity in model organisms to better represent human pathologies [8, 9].

The Diversity Outbred (DO) mouse model has emerged as a promising solution to these limitations. Unlike traditional inbred strains, DO mice are derived through random breeding of eight founder inbred strains. The result is a genetically heterogeneous mouse population, segregating for over 40 million single nucleotide polymorphisms [10, 11]. This model allows for specific hypotheses to be tested on a genetically diverse panel of mice that more closely mimics human populations while maintaining the ability to control for environmental factors in a laboratory setting. While a few studies have shown that genetic background influences neurogenesis, none have assessed how neurogenesis is impacted with age in a model with the extensive genetic diversity of the DO mouse [12–16]. The DO mouse model offers a unique opportunity to uncover insights that better reflect human genetic variation and its effect on neurogenesis.

To investigate how genetic diversity influences hippocampal neurogenesis with age, we quantified the formation of immature neurons in adult and aged Diversity Outbred mice, and benchmarked our findings against the commonly used inbred C57BL/6J strain. While both strains exhibit a similar decline in neurogenesis with age, DO mice display a significantly lower level of neurogenesis compared to C57BL/6J, even in young adults. To explore the contribution of a wild-derived strain to the reduction in neurogenesis in DO animals, we measured neurogenesis in the CAST/EiJ strain, and observed a similar reduction in new neuron formation, suggesting that this strain is partially responsible for reduced neurogenesis in DO animals.

## Materials and methods

### Animals and histology

All experiments were approved by the Institutional Animal Care and Use Committee at Brown University and The Jackson Laboratory. 6-month and 24-month male C57BL/6J (Strain #:000664) and Diversity Outbred mice (Strain #:009376) were obtained from The Jackson Laboratory through the Nathan Shock Center Pilot Grant Program. Mice were given ad libitum access to food and water and kept on a 12-h light/dark cycle.

Upon reaching 6 or 24 months of age, mice were anesthetized with Avertin and perfused with heparin/PBS followed by 4% paraformaldehyde. Brains were removed and post-fixed in 4% paraformaldehyde and dehydrated with a 30% sucrose gradient. After 2–3 days, the brains were embedded in Tissue-Tek® O.C.T. Compound and stored at −80 °C.

Adult (7-month) and aged (24-month) male CAST/EiJ brains were obtained from the Howell lab colony at The Jackson Laboratory. Mice were anesthetized with a ketamine/xylazine mixture and perfused with 1X PBS. The right hemisphere was fixed in 4% paraformaldehyde. The hemispheres were dehydrated and stored as described above.

All brains were cut into 40 µm coronal sections using a cryostat (Leica) and stored in cryoprotectant (30% ethylene glycol, 30% glycerol in 0.05 M phosphate buffer) at 4 °C.

### Immunohistochemistry

To visualize immature neurons in the dentate gyrus, every 6th section of hippocampal brain tissue was analyzed across the entire dentate gyrus. Sections were blocked in 10% Normal Donkey Serum (Jackson ImmunoResearch) and 1% Triton X-100 in 1X phosphate buffered saline (pH 7.4), then incubated with a rabbit polyclonal anti-Doublecortin primary antibody (1:500; Cell Signaling Technology) in 10% Normal Donkey Serum and 0.1% Triton X-100 overnight. Sections were washed in PBS and 0.01% Triton X-100 and incubated with an Alexa Fluor 488 donkey anti-rabbit secondary antibody (1:500; Invitrogen) for two hours. Sections were incubated in DAPI (5 mg/mL, 1:5000) in 1 × PBS for ten minutes and mounted onto glass slides with Mowiol mounting media for imaging.

### Image acquisition and analysis

Brain sections were imaged on a Zeiss Axiovert 200M Fluorescence Microscope for quantification analysis. Investigators were blinded to the identity of each sample while scoring. Doublecortin positive cells were manually counted and averaged per mouse brain. The data are reported as the mean ± SEM. The data were analyzed using a non-parametric Mann–Whitney U test. In all cases, *P* ≤ 0.05 was considered significant. All graphing and statistical analysis was performed in GraphPad Prism 9. Representative images for figures were taken on a Zeiss LSM980 Confocal Microscope.

## Results

### Lower neurogenesis in Diversity Outbred mice relative to C57BL/6J mice

**Fig. 1:**
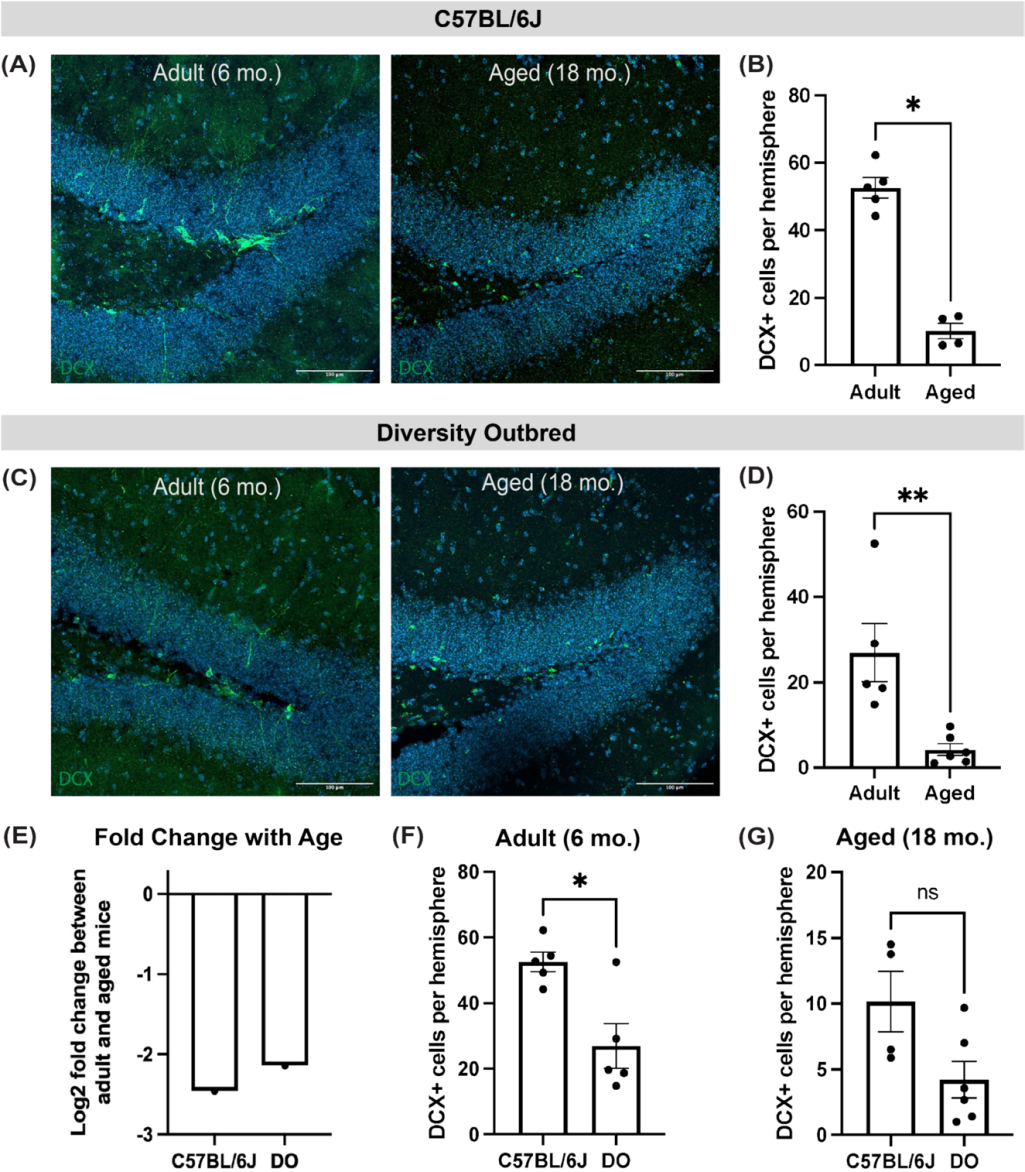
Neurogenesis is impaired with age in the Diversity Outbred mouse model and reduced relative to C57BL/6J inbred mice. **A** Representative confocal microscopy images of the dentate gyrus (DG) of adult (6 month) and aged (18 month) C57BL/6J mice stained for Doublecortin (DCX) (green). **B** Quantification of the numbers of the average DCX-positive cells in the DG of adult and aged C57BL/6J mice (Mann Whitney U test, *p = 0.0159, *n* = 5 adult mice, *n* = 4 aged mice). **C** Representative confocal microscopy images of the dentate gyrus of adult (6 month) and aged (18 month) Diversity Outbred mice stained for DCX (green). **D** Quantification of the average DCX-positive cells in the DG of adult and aged Diversity Outbred mice (Mann Whitney test, **p = 0.0043, *n* = 5 adult mice, *n* = 5 aged mice). **E** Log2 fold change of the average DCX-positive cells between adult and aged C57BL/6J mice and adult and aged DO mice. **F** Average DCX-positive cells in the DG of adult C57BL/6J and DO mice (Mann Whitney test, *p = 0.0317, *n* = 5 adult C57BL/6J mice, *n* = 5 adult DO mice). **G** Average DCX-positive cells in the DG of aged C57BL/6J and DO mice (Mann Whitney test, *p* = 0.1143, *n* = 4 aged C57BL/6J mice, *n* = 6 aged DO mice)

We first validated that the formation of new neurons in the subgranular zone of the hippocampus decreases with age in 6 month and 18 month old C57BL/6J mice using the immature neuron marker Doublecortin (DCX). As previously observed [3, 17], the number of DCX-positive cells was significantly decreased in the aged C57BL/6J mice compared to the adult mice (Fig. 1a, b). We next assessed whether this age-associated decrease in neurogenesis could also be observed in the DO mouse model. Indeed, the number of DCX-positive cells declined with age in DO mice (Fig. 1c, d). These results demonstrate that the Diversity Outbred mouse model exhibits a similar trend in age-associated decline in neurogenesis as the traditional inbred C57BL/6J strain, with a comparable fold change with age between the adult and aged mice of both strains (Fig. 1e).

We next assessed whether genetic background influences the formation of new neurons in adult and aged mice. Interestingly, when we compared the number of immature neurons in the subgranular zone of adult C57BL/6J and DO mice, there was a significant decrease in DCX-positive cells in the DO mice relative to the C57BL/6J inbred background (Fig. 1f). This trend was also observed in the aged C57BL/6J and DO mice, but did not reach significance due to a limited number of animals (Fig. 1g). These results suggest that the DO mouse model exhibits a reduced overall level of neurogenesis compared to the traditional inbred mouse strain.

### Neurogenesis is significantly reduced in CAST/EiJ mice compared to C57BL/6J mice

**Fig. 2:**
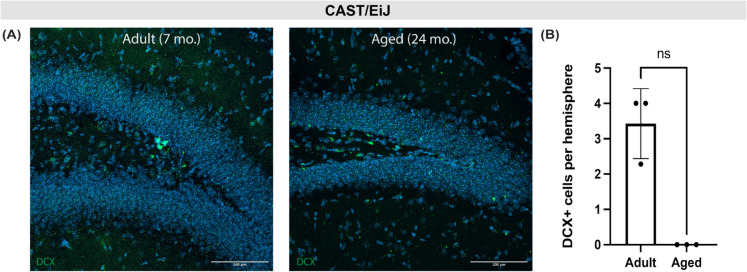
Low neurogenesis in CAST/EiJ mice is dramatically reduced with age. **A** Representative confocal microscopy images of the dentate gyrus of adult (7-month) and aged (24-month) CAST/EiJ mice stained for DCX (green). **B** Average DCX-positive cells in the DG of adult and aged CAST/EiJ mice (Mann Whitney test, *p* = 0.1000, *n* = 3 for both age groups)

We next assessed whether the decrease in neurogenesis we observed in the DO mice is phenotypically consistent with one of the eight founder strains. CAST/EiJ mice were identified as a candidate for driving a decrease in neurogenesis due to reports of altered circadian rhythms, which can negatively impact adult neurogenesis [18–20], and a previous study in which CAST/EiJ mice were observed to have lower new neuron formation in young adult mice (8 week) compared to C57BL/6J mice [16]. Indeed, when we stained adult and aged CAST/EiJ brain tissue for DCX, the number of DCX-positive cells in the dentate gyrus were decreased in comparison to both the C57BL/6J mice and DO mice (Fig. 2a, b), but the decrease with age did not reach significance due to a limited sample size. These results suggest that the genetic contribution from the CAST/EiJ may be partially contributing to the decrease in neurogenesis observed in the DO mouse model.

**Fig. 3:**
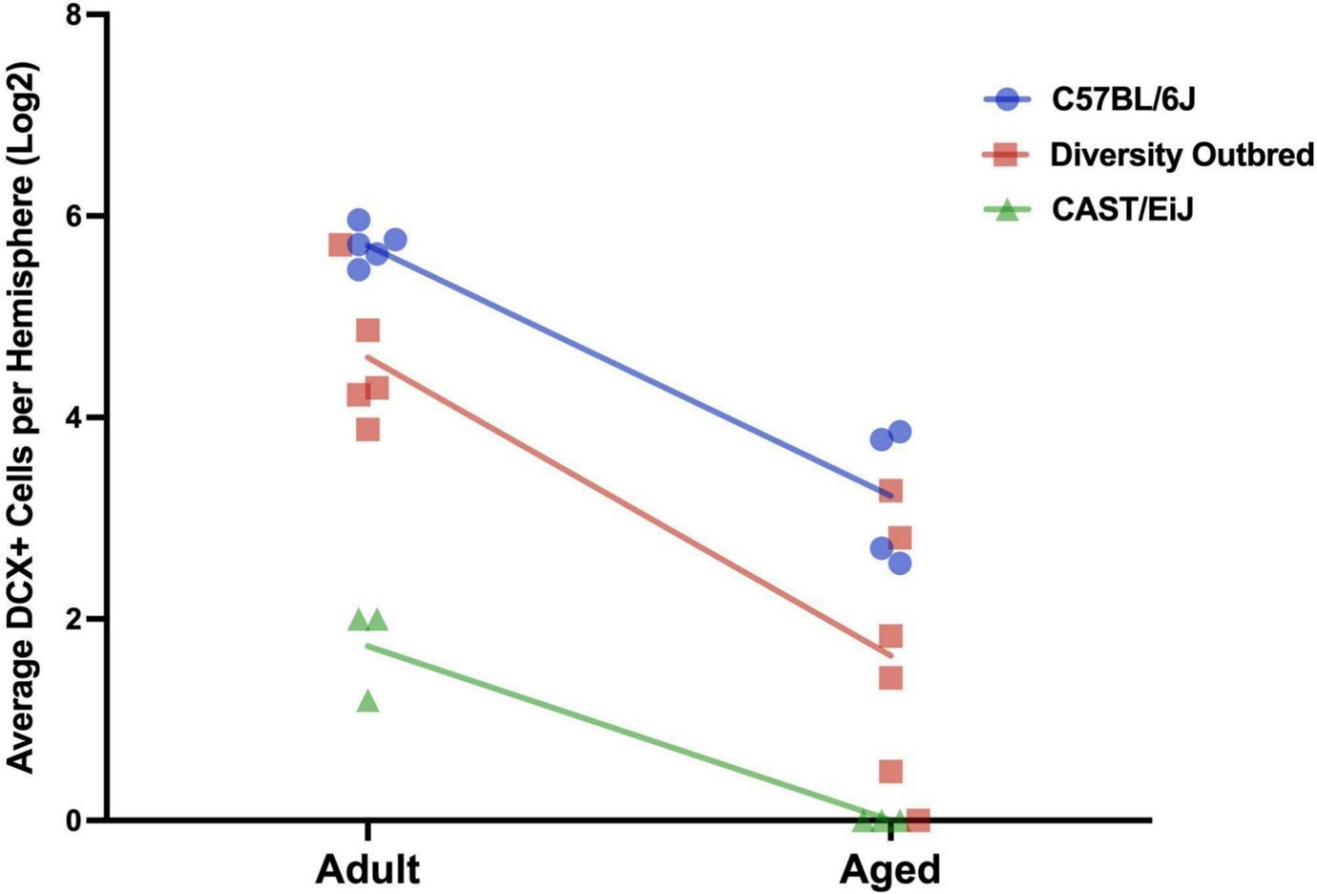
Neurogenesis declines with age at a similar rate between C57BL/6J, Diversity Outbred, and CAST/EiJ mice. The average DCX-positive cells per hemisphere between C57BL/6J, Diversity Outbred, and CAST/EiJ mice demonstrate a similar rate of decline from the adult to the aged timepoint

Lastly, we compared the rate of decline of neurogenesis across the DO, C57BL/6J and CAST/EiJ lines. All three strains show a similar rate of decline in neurogenesis with age, with the C57BL/6J model displaying the highest baseline level of neurogenesis and the CAST/EiJ displaying the lowest (Fig. 3).

## Discussion

In this study, we investigated the extent to which neurogenesis is impaired with age in a genetically diverse mouse strain, and benchmarked it against the commonly used C57BL/6J inbred strain. This analysis revealed reduced neurogenesis in adult and aged Diversity Outbred mice compared to the inbred C57BL/6J mouse strain. This decrease may be partially driven by the CAST/EiJ founder strain. Interestingly, the rate of decline of neurogenesis with age was similar in all three strains, suggesting that similar mechanisms may underlie the age-associated loss of neurogenesis in the distinct lines.

Previous studies that have compared levels of neurogenesis in mice with differing genetic backgrounds have reported similar findings, with C57BL/6J mice displaying increased proliferating Ki67-positive cells and DCX-positive cells compared to ICR (Institute for Cancer Research) and BALB/c mice [12], which are strains commonly used in cancer, immunology, and infectious disease studies. Young adult (8 week) C57BL/6J mice have also been shown to harbor a higher number of proliferating neural progenitors (BrdU-positive cells) and new mature neurons (BrdU-positive NeuN-positive) compared to 12 different isogenic strains [16]. Together with this study, these results indicate that the overall number of proliferating cells and newly generated neurons in C57BL/6J mice is higher than that of other mouse strains. C57BL/6J mice have also been classified as “good learners” [21, 22], which is likely partly attributed to their increased hippocampal neurogenesis. These results, combined with the findings of our study, demonstrate the powerful influence of genetic background on neurogenesis and underscore the need for genetic diversity in the lab setting to ensure translatability to humans.

This is the first study to show that the Diversity Outbred line exhibits lower levels of neurogenesis relative to the common inbred C57BL/6J mouse strain. Strikingly, adult DO mice display only slightly higher levels of neurogenesis than aged C57BL/6J mice. Previous studies have shown that DO mice fail to display improved late-life memory in response to caloric restriction or intermittent fasting, which are established methods for enhancing cognitive performance in C57BL/6J mice [23, 24]. Caloric restriction has also been shown to increase neurogenesis in the aging rodent brain, and lower baseline neurogenesis with age in the DO model may contribute to this failure to recapitulate the cognitive improvement observed in C57BL/6J mice.

The wild-derived CAST/EiJ mouse strain is one of the eight founder strains incorporated in the Diversity Outbred mouse model. Based on our work and others, CAST/EiJ mice display a low baseline level of neurogenesis relative to other mouse strains [16], and it is possible that the genetic contribution from the CAST/EiJ strain is partially responsible for the decreased number of DCX-positive cells observed in the DO model. CAST/EiJ display impaired circadian rhythms, and circadian rhythm dysfunction impacts hippocampal function [19, 20], providing a possible explanation for low neurogenesis in this strain. Nevertheless, CAST/EiJ mice were also observed to be high responders in a voluntary exercise paradigm; exercise-induced neurogenesis was strongly increased in these mice compared to other strains tested, including C57BL/6J. Thus, baseline levels of neurogenesis do not represent maximum neurogenic capacity. In the future, further work that probes the mechanism by which the CAST/EiJ strain influences the phenotypes observed in the DO model is necessary to fully elucidate the molecular mechanisms responsible for the difference in neurogenesis.

This study has a few limitations related to the mouse cohorts that should be considered. First, our analysis was limited to male mice, which may restrict the generalizability of our findings to females. Given well-documented sex differences in brain aging and longevity, including both sexes in future studies is essential. Additionally, in some cases, the number of mice per experiment was relatively low. The inclusion of additional animals will be important for strengthening the robustness and interpretability of future findings. Lastly, a time-course analysis would be useful to fully elucidate the trajectory of decline in neurogenesis in the different strains.

Follow-up experiments incorporating additional neurogenesis markers such as BrdU, Ki67, and NeuN will better determine at which stage the neurogenic lineage is altered in genetically diverse mice. For example, the overall stem cell pool could be lower in the dentate gyrus, the neural stem cells could be delayed in activation, or there could be increased death during maturation. Furthermore, examining the other founder strains that contribute to the DO model could clarify their individual roles in determining the neurogenesis levels. Deeper mechanistic work into how these individual strains influence neurogenesis in the DO model will provide better insight into the genetic regulation of this process.

In conclusion, this study highlights the significant impact of genetic diversity on hippocampal neurogenesis, demonstrating that the Diversity Outbred mouse model exhibits reduced baseline neurogenesis but a similar age-related decline in new neuron formation compared to the C57BL/6J strain. Baseline differences in neurogenesis may potentially be influenced by the CAST/EiJ founder strain. The Diversity Outbred mouse model offers a valuable and more representative model for studying neurogenesis, as its genetic diversity provides a closer approximation of human genetic variation, enhancing the translational relevance of experiments conducted in the model.
